# Alpha-gal syndrome and delayed anaphylaxis after ingestion of red meat: A case report 

**DOI:** 10.5414/ALX02394E

**Published:** 2022-12-15

**Authors:** Lea Caron, Valeria G.R. Ortolani, Eleonora Bono, Christian P. Ratti, Enrico Iemoli

**Affiliations:** Allergy and Clinical Immunology Unit, ASST FBF Sacco, Milan, Italy

**Keywords:** α-gal syndrome (AGS), anaphylaxis, food allergy

## Abstract

α-gal syndrome (AGS) is caused by the intake of products containing α-gal (galactose-α-1,3-galactose) like mammalian meat. Over the last decade, scientific literature about AGS has been increasing, but the true burden of cases is still unknown [1, 2]. In the USA (University of Virginia Allergy Clinic), the number of confirmed cases of AGS was 24 in 2009 [3] and increased to 34,000 in the entire USA by 2019 [4]. As shown in surveys, in Italy AGS is present throughout the country [5]. The literature suggests that a previous tick bite can cause AGS, but in our case it was not possible to demonstrate this association as the patient did not recall any tick bite, even in childhood. After eating red meat, a 56-year-old male patient had developed symptoms such as a generalized urticaria, diarrhea, and faintness, requiring admission to the Emergency Department. The diagnosis was verified using blood CAP-FEIA test and prick-to-prick test. After completing the diagnostic process, we provided the patient with emergency therapy, and auto-injectable adrenaline was prescribed. Despite the diagnosis, the patient ate red meat once again which resulted in severe urticaria 2 hours after the meal, requiring a second visit to the Emergency Room. Now the patient is under follow-up at our Department of Allergy and Clinical Immunology.

## Introduction 

α-gal syndrome (AGS), also called “α-gal allergy”, “red meat allergy”, or “red meat allergy caused by tick bite”, is a severe, potentially life-threatening, allergic reaction [2, 3]. 

AGS is caused by the intake of red meat or products containing α-gal (galactose-α-1,3-galactose), a disaccharide which the human immune system encounters, in some cases, through the bite of a tick, an arthropod vector. In certain cases, the patient does not know that a tick bit them, and, in addition, red meat is not generally considered as a common allergen, further complicating the diagnostic process. Hypersensitivity results from the production of specific IgE against α-gal (sIgE-α-gal) [1, 2, 6]. This can occur in two forms: an immediate hypersensitivity or a delayed hypersensitivity. In both cases, anaphylaxis may result, either immediately or delayed. AGS is a potential cause of idiopathic anaphylaxis and should be considered, and therefore excluded, especially when the anaphylactic reaction is delayed [6]. AGS is a quite newly discovered syndrome, in addition to being a potential allergen hidden in prepared foods (Table 1). Adverse events related to AGS can occur with the same or different entity and may vary depending on the presence of cofactors (Table 2). 

## Aims 

We aimed to contribute to the broadening of knowledge of the AGS and its characteristics. Another primary objective is to focus on those aspects that may be helpful or misleading to the specialist, based on our experience. 

## Case presentation 

The patient is a 56-year-old Caucasian male, known to our allergy center since 2018 for asthma and respiratory allergy (*Dermatophagoides pteronyssinus* and *Cupressus arizonica*). 

In October 2021, during the annual check-up, allergists were informed about an episode of general malaise, which had occurred in the previous month and required an admission to the Emergency Room (ER). 

The patient suffered from diffuse urticaria and other systemic symptoms, such as diarrhea and faintness. In the ER, paramedics detected hypotension (blood pressure (BP) 90/50 mmHg, heart rate (HR) of 64 bpm), an extensive skin reaction, without dyspnea or other symptoms. Antihistamines and corticosteroids were administered, and blood volume was restored, until the symptoms slowly resolved. Arterial blood gas analysis showed a slight hypokalemia; thus, the patient was treated with potassium chloride on the next day. Doctors interviewed him regarding the events that preceded the reaction, but they did not identify any possible triggers at first glance. He had no dinner because of a heavy lunch with spinach and ricotta ravioli, pork, nectarine, and perhaps wine. He also reported that he tested and tolerated all these foods after the reaction, except for ravioli (which he did not eat again after the anaphylaxis), but later tolerated the pasta and ricotta. At the same time, he refused symptoms referable to infections, any medication (even in the previous days), insect bites, or physical activity. The reasons for the suspected anaphylactic episode were not determined at this time. Due to the latency of the onset of symptoms, a food allergy as the cause of the reaction was considered very unlikely. To complete the diagnostic workup, we performed blood CAP-FEIA test with the following findings: baseline tryptase 5.7 µg/mL; total IgE 229 kU/L; negative for allergen extracts of egg, yolk, peanuts, soybeans, hazelnuts, almonds, peach, walnut, celery, parsley, cashew, pistachio, pine nuts, black pepper, macadamia nut; negative for recombinant allergens Pru p1, Pru p4, Pru p3, Gal d1, Gal d2, Gal d3, Gal d4, Fel d1, Fel d2. There was instead a positive response to allergen extracts from pork meat (2.46 kU/L) and spinach (0.11 kU/L), and finally to cat epithelium (0.14 kU/L). CAP-FEIA was again prescribed for α-Gal, Fel d2, beef allergen extracts, and we found positivity to sIgE-α-gal with a value of 7.7 kU/L and beef (3.4 kU/L). The patient was contacted immediately to further clarify his patient history, and he stated that he had recently eaten pork sausages with no new adverse reactions. We also decided to perform prick-to-prick tests with red meat, and findings were positive for raw beef, cooked beef, raw and cooked pork (Table 3). We diagnosed AGS, and we tried to explain to the patient, with as much detail as possible, what it implies, which foods to consume and which ones better to avoid, with particular attention to the cofactors. Emergency therapy and self-injectable adrenaline were prescribed and explained, providing all the instructions necessary to use it appropriately. 

The patient, while understanding the severity of the previous reaction, contacted us after a couple of weeks reporting that he had to go to the ER again after eating lasagna containing meat. From the documentation released by the ER we learned that the patient had developed a strong rash (urticaria) spread all over the body and diarrhea 2 hours after the meal. Vital signs were normal (BP 120/70 mmHg, HR 82 bpm). The patient, conscious of the diagnosis, quickly went to the hospital, and the urticaria resolved completely after administration of antihistamine and cortisone at the ER and later at home. 

## Discussion 

There have been more and more AGS surveys in the literature over the last decade, but the true burden of cases is still unknown [2]. The allergen responsible for hypersensitivity is a disaccharide rather than a protein [5, 9]. We were supported by advanced laboratory techniques which can detect specific antigens to uncommon recombinant allergens such as α-gal. 

In AGS, the onset of symptoms generally occurs from 3 to 6 hours [6, 7, 10] after the ingestion of the meal. Cofactors can determine an interindividual variability and different reactions even in the same individual and are thus confounding factors.

The reaction could be milder if the patient takes the allergen in smaller doses or depending on the form of the allergen intake (i.e., cooked/raw/processed) and on its organoleptic characteristics (i.e., low-fat meat, fat meat, organ meats). Alcohol ingestion may have been an important cofactor in the reaction [1, 10]. 

The etiopathogenesis of AGS is strongly related to tick bites [2, 4], but, in our case, it was not possible to find this association. 

In fact, the patient had no memory of ever being bitten by a tick in the past, even if he remembered that his brother was bitten by a tick during his childhood in a country house. Recently, our patient had spent some time in the mountains. 

Prick-to-prick tests have been confirmed as a reliable and reproducible diagnostic method [1, 3, 8]. The positive findings in the CAP-FEIA and prick-to-prick tests confirmed the clinical suspicions of the allergist, but above all they helped the patient to understand the events he experienced. 

Despite this, a few days after the diagnosis, the patient ate red meat again, in a lasagna. The reaction occurred with minor intensity than before, and the patient was careful to follow our instructions. However, unlike in the previous episodes, the patient had at least tried to avoid co-factors. In fact, the food was mostly cooked and low in fat, and the amount consumed was smaller (Table 2). 

Unfortunately, it is improbable to find a threshold that can be defined as safe in allergic diseases [1, 9]. AGS is potentially life-threatening, and for this reason it is extremely important to advise the patient to follow a controlled diet that avoids as much as possible the intake of red meat, which responsible for reactions [1]. If an AGS patient has problems with the strict diet, a nutritionist should be consulted so as not to impair the quality of life too much. However, skin tests for food allergens derived from animals (such as milk or meat antigens of other species) could be useful and should be combined with a targeted patient history, both to reassure the patient about the possibility of taking other types of animal derivatives and to avoid their exclusion from the patient’s diet. In fact, AGS does not force the patient to eliminate all foods of animal origin but only those that have not been tolerated and those that can potentially give the same reaction (in our case all types of red meat). We also recommend AGS patients to avoid red meat even in cooked or processed form, as this allergen can withstand chemical or thermal treatments. Our patient tolerates milk, eggs, and other derivatives, so we did not suggest avoiding them, just like Platts-Mills et al. [6] have suggested. If patients are in areas where ticks may be present, we recommend to prevent tick bites that can trigger another allergic reaction (for example by wearing light-colored clothes or applying specific tick repellents, such as permethrin) [7, 11]. 

α-gal is a “hidden allergen”, the patient must be informed about that and be instructed to understand food labels, with particular attention to the preservative E407 and the gelatin present in confectionery preparations and in some drugs (Table 1). The patient must be equipped with an emergency therapy (including self-injectable adrenaline) with adequate information about it and the recommendation to perform periodic training at home (using multimedia contents, such as videos regarding the use of the adrenaline pen). The patient should be retrained under the supervision of an allergist whenever they come to the clinic. The objective is to support patients and to enable them to monitor the signs and symptoms in a professional manner (also focusing on the aspect that signs and symptoms may occur even hours after eating) and how to manage their lives with AGS. We also recommend educating patients to always carry the clinical documents released by the allergist (allergy pass). This documentation is of fundamental importance in the event of accidental ingestion of the allergen to allow the healthcare staff of any hospital to be aware of the diagnosis of AGS and therefore of its management. We believe that the new episode of adverse reaction that occurred in our patient will cause him to watch out for α-gal-containing foods and further confirms the diagnosis AGS.

In 1 year monitoring the patient, he had no more episodes of meat allergy. He periodically performs checkups, and now he is aware of the importance of avoiding these types of foods. 

During the last interview, he reported that he accidentally ingested a preparation made from chemically processed pork fat, without symptoms. It is currently not possible to determine whether and when the patient will be able to tolerate the meat again. The patient seems to tolerate meat in certain situations, evidence that suggests a decisive role of the cofactors. However, for his safety, it was decided collegially to avoid the ingestion of all types of red meat for now. 

## Conclusion 

AGS should be suspected in a patient with delayed anaphylaxis, especially if there is a tick bite and meat intake, even hours before the reaction, in his clinical history in his clinical history. For diagnosis, the CAP-FEIA serologic method was useful as a preliminary confirmation of clinical doubt; however, if unavailable, or in combination, the prick-to-prick test is essential for further certainty about the possible cause of anaphylaxis. It is useful to carry out the prick-to-prick test with uncooked and cooked meat, as in our case the patient was positive for both kinds of preparation, which confirms that the allergen is not destroyed during cooking. 

Even if a small amount of meat was accidentally ingested and tolerated afterwards, in our experience, reactions can recur unpredictably and with a more worrying and more rapid onset, especially in the presence of cofactors. For this reason, it is important to support the patient and help him to fully understand the risks associated with AGS and how to prevent the occurrence of further adverse reactions. α-gal could be included in medicines; for this reason, the patient should inform their physician about AGS before any treatment. 

## Funding 

The authors did not receive funding for this paper. 

## Conflict of interest 

The authors have no conflict of interest to declare. 

**Table 1.** Sources of α-gal.

Meat and internal organs of non-primate mammals	Beef, pork (even hog casings for sausages [6]), elk, reindeer, lamb, but also small mammals such as squirrels or beaver
Derivatives of non-primate mammals.	Milk and milk products [10]
Industrially processed products	Gelatins [10] (e.g., carrageenan, E407), thickening agents, sauces, candies, ready-made.
Drugs (immediate reactions, especially if administered parenterally)	Monoclonal antibodies (e.g., cetuximab [1]), vaccines (e.g., MMR, Zostavax) [4], recombinant coagulation factor substitutes), plasma expanders [5], animal-derived pancreatic enzymes [1], anticoagulants (e.g., heparin [1]), magnesium stearate [6] capsules, syrups, nasal sprays**,** antidotes for snake venom [1, 3]
Biological prostheses	Cardiovascular valve of animal origin (pigs or bovines) [1]

**Table 2.** Additional risk factors and cofactors.

Recurring tick bites [11]	
Recurring hymenopteran stings [1]	
Alcohol [4, 6, 10]	
Exercise/workout [4, 6]	
Concomitant intake of drugs	Non-steroidal anti-inflammatory drugs (NSAIDs) [6, 10]
Kind of meat	Organ meats > α-gal content [10], fatty meat > risk of severe reactions [10]
Age	> 50 years, increases the risk of AGS [11]

**Table 3.** Main test results: skin tests and sIgE from CAP-FEIA.

	Skin prick test	Prick-to-prick test	sIgE
Allergen	Wheal (mm)	Wheal (mm)	kU/L
α-gal	NT	NT	7.7
Pork	NT	raw = 5	2.46
	cooked = 3		
Beef	NT	raw = 10	3.4
	cooked = 5		
Cat epithelium	4	NT	0.14
Spinach	NT	NT	0.11
Peach, recombinant (rPru p1, rPru p3, rPru p4)	NT	NT	Negative
Egg, recombinant (Gal d1, Gal d2, Gal d3, Gal d4)	NT	NT	Negative
Cat, recombinant (Fel d1, Fel d2)	NT	NT	Negative
Milk, recombinant (nBos d4, nBos d5, nBos d8)	NT	NT	Negative

s-IgE = specific IgE; NT = not tested.
